# A Case of Lymphocytic Infundibulo-neurohypophysitis Exhibiting Spontaneous Regression

**DOI:** 10.1210/jcemcr/luad020

**Published:** 2023-02-27

**Authors:** Masashi Hasebe, Kimitaka Shibue, Sachiko Honjo, Akihiro Hamasaki

**Affiliations:** Department of Diabetes and Endocrinology, Medical Research Institute, Kitano Hospital, PIIF Tazuke-Kofukai, Kita-ku, Osaka 530-8480, Japan; Department of Diabetes and Endocrinology, Medical Research Institute, Kitano Hospital, PIIF Tazuke-Kofukai, Kita-ku, Osaka 530-8480, Japan; Department of Diabetes and Endocrinology, Medical Research Institute, Kitano Hospital, PIIF Tazuke-Kofukai, Kita-ku, Osaka 530-8480, Japan; Department of Diabetes and Endocrinology, Medical Research Institute, Kitano Hospital, PIIF Tazuke-Kofukai, Kita-ku, Osaka 530-8480, Japan

**Keywords:** anti-rabphilin-3A-antibodies, central diabetes insipidus‌, lymphocytic infundibulo-neurohypophysitis, pituitary gland, pituitary stalk, primary lymphocytic hypophysitis

## Abstract

Lymphocytic infundibulo-neurohypophysitis (LINH) is a rare autoimmune inflammatory process that selectively affects the neurohypophysis and the pituitary stalk, typically presenting with central diabetes insipidus (CDI). LINH is considered underdiagnosed because the definitive diagnosis requires invasive pituitary surgery with a high risk of complications. We present a case of CDI resulting from LINH, which was treated with conservative management, eschewing both glucocorticoid treatment and pituitary surgery. At presentation, the hormonal assessment indicated the presence of CDI without anterior pituitary dysfunction. Magnetic resonance imaging revealed stalk thickening without a posterior pituitary bright spot, and anti-rabphilin-3A antibodies were positive in serum. Collectively, we made a diagnosis of LINH. Considering that the patient did not exhibit any symptoms of mass effect, we chose conservative treatment with desmopressin acetate. One year later, the stalk thickening regressed spontaneously without surgical or glucocorticoid treatment, although the posterior pituitary bright spot remained absent, and CDI did not improve. The inflammatory process of LINH is mostly self-limited and recovers spontaneously, whereas life-long desmopressin treatment may be required because of pituitary stalk fibrosis and atrophy. Our case highlights the importance of noninvasive diagnosis and careful follow-up in preventing unnecessary interventions for patients with LINH.

Lymphocytic infundibulo-neurohypophysitis (LINH) is a rare autoimmune inflammatory disorder that selectively affects the posterior pituitary and the pituitary stalk, resulting in swelling of the posterior pituitary and stalk on neuroimaging [1]. This disorder typically presents with central diabetes insipidus (CDI) without anterior pituitary dysfunction or mass effects [2]. The definitive diagnosis of LINH requires a pituitary biopsy using a transsphenoidal approach with histopathological analysis; however, the biopsy is limited to specialized settings because of its invasiveness and potential adverse events [3]. Therefore, LINH is often underdiagnosed, despite being considered a major cause of idiopathic CDI [2]. Although the best management strategy in LINH remains incompletely understood, the inflammatory process is mainly self-limited. It does not require high-burden treatment in most cases [2]. Hence, accurate diagnosis and careful follow-up with less invasive strategies, at least initially, are critical to prevent unnecessary interventions such as pituitary surgery or glucocorticoid (GC) treatment.

Here, we describe a case of CDI that occurred in a young Japanese woman, apparently without predisposing factors. We noninvasively diagnosed LINH as the etiology of her CDI by performing neuroimaging and detecting serum anti-rabphilin-3A antibodies, a specific biomarker of LINH [4]. Following the diagnosis, we observed the spontaneous regression of the pituitary stalk enlargement over time with conservative management, determining the self-limited inflammatory process of LINH. Our case highlights the relevance of appropriate clinical, hormonal, and radiological evaluation in suspected cases of LINH for improving the overall clinical outcome.

## Case Presentation

A 31-year-old woman was referred to our hospital with symptoms of polyuria and polydipsia. She noted a marked increase in urine output and water consumption (both more than 10 L daily) in the past year. She had no remarkable medical history, including brain diseases and psychiatric disorders, and no history of pregnancy. She was not taking any medication, including immunomodulating agents. On physical examination, she showed moderately diminished skin turgor and dry mouth. She did not have symptoms of mass effects such as visual disturbance or headache. Laboratory investigations revealed low urinary osmolality (53 mOsm/kg) and low specific gravity (1.001), whereas other laboratory studies were within normal limits. These findings made us consider the possibility of diabetes insipidus, and we performed hypertonic-saline (5%) infusion test and vasopressin challenge test. The hypertonic-saline infusion test revealed a severely blunted arginine vasopressin response (Table 1). In contrast, the urine osmolality increased following the subcutaneous injection of Pitressin at a dose of 5 U (Table 2). Based on these findings, we diagnosed CDI as the cause of her symptoms, followed by a differential diagnosis of its etiology.

**Table 1.** Results of 5% hypertonic-saline infusion test

	Reference range for basal value	0 min	30 min	60 min	90 min	120 min
Serum osmolarity, mOsm/kg	275-290	288.0	296.0	303.0	309.0	312.0
Serum sodium, mEq/L	138-145	144.0	148.0	152.0	156.0	156.0
Plasma arginine vasopressin, pg/mL	<2.8	0.4	0.4	0.4	0.4	0.5

**Table 2.** Results of arginine vasopressin challenge test

	Reference range for basal value	0 min	30 min	60 min
Serum osmolarity, mOsm/kg	275-290	296.0	296.0	297.0
Urine osmolarity, mOsm/kg	50-1300	95.0	305.0	440.0

## Diagnostic Assessment

**Figure 1.**
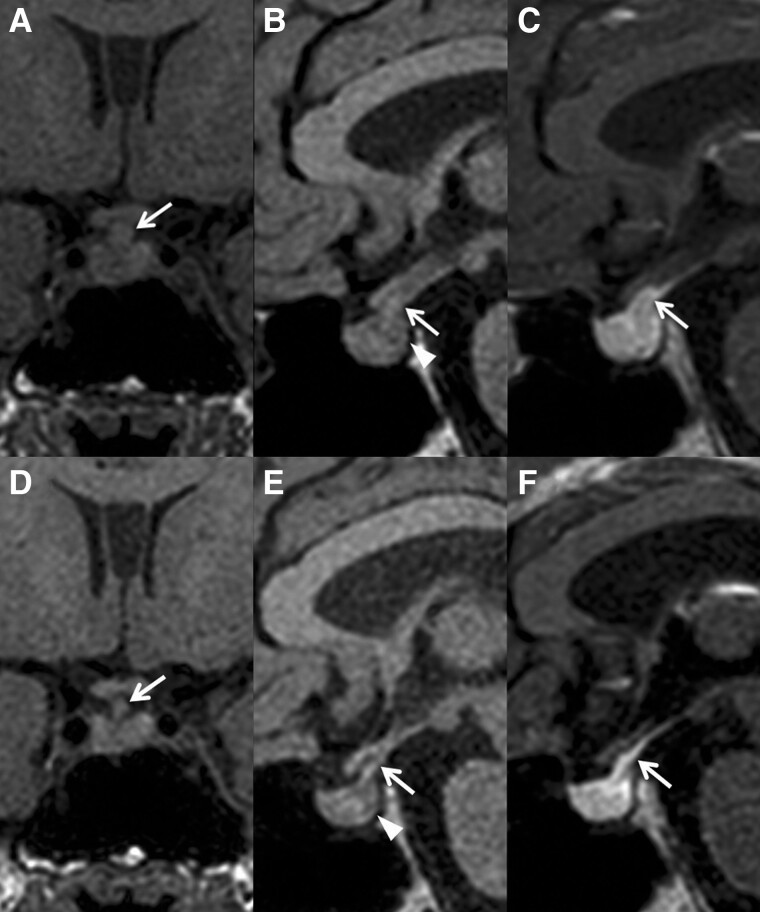
Magnetic resonance imaging of hypothalamic-pituitary regions. (A-C) Thickening of the pituitary stalk (arrows) and the absence of the posterior pituitary bright spot (arrowhead) at initial presentation (A, B: noncontrast T1-weighted; C: contrast-enhanced). (D-F) Regression of the pituitary stalk thickening (arrows) and the persistent lack of the posterior pituitary bright spot (arrowhead) 1 year after presentation (D, E: noncontrast T1-weighted; F: contrast-enhanced).

Noncontrast magnetic resonance imaging (MRI) of hypothalamic-pituitary regions demonstrated stalk thickening (Fig. 1A, B, arrows) with the absence of a posterior pituitary bright spot on the T1-weighted image (Fig. 1B, arrowhead). After administering gadolinium contrast medium, the enlarged stalk and the entire pituitary were rapidly enhanced (Fig. 1C). These findings suggested the presence of LINH as a cause of CDI [1, 2]. Because systemic diseases such as IgG4-related hypophysitis, malignant lymphoma, Wegener granulomatosis, sarcoidosis, germ cell tumor, fungal infection, and tuberculosis can mimic LINH both in clinical and radiographic findings [1, 5], we measured the following biomarkers: IgG4, soluble IL-2 receptor, anti-neutrophil cytoplasmic antibody, angiotensin-converting enzyme, β human chorionic gonadotropin, α-fetoprotein, β-D glucan, and T-SPOT. Consequently, all those markers were found to be within their normal limits or negative. Additionally, anti-rabphilin-3A antibodies, a specific biomarker of LINH [4], were detected in the serum by western blotting. Altogether, these findings established the clinical diagnosis of LINH. We also evaluated anterior pituitary function by administering GnRH (0.1 mg), thyrotropin-releasing hormone (0.2 mg), corticotropin-releasing hormone (100 μg), and GH-releasing peptide-2 (100 μg). The basic hormonal secretion and responses to the stimulation of the anterior pituitary gland were normal (Tables 3 and 4), indicating the absence of anterior pituitary involvement. Considering that LINH is an autoimmune disease that selectively affects the posterior lobe of the pituitary and the pituitary stalk [1, 2], the results of the anterior pituitary function tests were consistent with the clinical diagnosis of LINH.

**Table 3.** Results of GnRH (0.1 mg), thyrotropin-releasing hormone (0.2 mg), and corticotropin-releasing hormone (100 μg) stimulation test

	Reference range for basal value	0 min	30 min	60 min	90 min	120 min
Serum thyrotropin, μIUmL	0.6-4.2	0.8	8.8	5.8	3.9	2.6
Plasma adrenocorticotropin, pg/mL	7.4-55.7	11.5	129.0	59.3	29.6	6.9
Serum cortisol, μg/mL	3.7-19.4	16.5	32.9	31.5	21.8	16.5
Serum LH, mIU/mL	0.5-15.0	16.2	150.9	141.1	129.9	126.8
Serum FSH, mIU/mL	1.3-24.0	4.6	15.3	16.7	18.1	18.5
Serum prolactin, ng/mL	3.5-32.7	10.5	55.7	35.9	23.8	22.3

**Table 4.** Results of GH-releasing peptide-2 (100 μg) stimulation test

	Reference range for basal value	0 min	15 min	30 min	45 min	60 min
Serum GH,ng/mL	0.1-9.9	0.3	33.8	24.7	15.9	10.1

Although we proposed transsphenoidal pituitary biopsy to the patient for the definitive diagnosis, the patient refused the procedure because of its invasiveness. We therefore decided to undertake conservative management and careful follow-up with serial hormonal and MRI evaluations.

## Treatment

Treatment began with oral desmopressin acetate (60 μg twice daily). Subsequently, the patient’s daily urine output decreased to less than 3 L daily, and her polydipsia also drastically improved. We did not perform either GC treatment or surgical decompression, considering her lack of severe or progressive mass effect symptoms and the anterior pituitary dysfunction because conservative management is recommended in patients with mild symptoms or signs [3].

## Outcome and Follow-up

During the 1-year follow-up after the initial diagnosis, the patient did not develop any symptoms associated with mass effects, such as headache or visual field defects. Moreover, she did not develop symptoms or signs of anterior pituitary dysfunction, and routine hormonal assessment of the anterior pituitary function remained normal. Although her CDI did not improve and desmopressin treatment was continuously required, she remained free from pituitary surgery and GC treatment. Notably, 1 year after presentation, she underwent a follow-up MRI that demonstrated the spontaneous regression of the pituitary stalk enlargement, indicating the self-limited inflammatory process of LINH (Fig. 1D-1F, arrows). On the other hand, the posterior pituitary bright spot remained absent, consistent with the clinical course of persistent CDI (Fig. 1E, arrowhead).

## Discussion

Primary lymphocytic hypophysitis (PLH) is a rare autoimmune disorder characterized by inflammation of the pituitary or neuroinfundibulum. However, because of the nonspecific symptoms and the difficulty in making a definitive diagnosis, it is considered to be underdiagnosed [5]. According to the affected locations, PLH is subdivided into 3 forms: (1) lymphocytic adeno-hypophysitis; (2) LINH; and (3) lymphocytic pan-hypophysitis [5]. Although the gold standard for diagnosing PLH is histopathological examination of a pituitary sample, it is not feasible to routinely perform pituitary biopsy because of the potential adverse complications, including further deterioration of the pituitary function and mortality. Therefore, it is critically important to establish a presumptive diagnosis based on noninvasive investigations while excluding other etiologies.

In LINH, lymphocytes selectively infiltrate the neurohypophysis, leading to CDI and swelling of the posterior pituitary and the pituitary stalk [1]. As with other subtypes of PLH, the definitive diagnosis of LINH can be established by invasive pituitary biopsy; however, it can be avoided through appropriate clinical, biochemical, and radiological assessment as in the present case. In particular, MRI scan of hypothalamic-pituitary regions plays a pivotal role in making the clinical diagnosis without the need for a pituitary biopsy [1, 2]. The absence of the posterior pituitary bright spot on the noncontrast T1-weighted MRI scan indicates a defect in vasopressin storage, typical of LINH. However, the finding is also seen in the healthy elderly population [6]. Nevertheless, the lack of a bright spot presented with the stalk thickening, as in our patient at the initial presentation, is highly relevant to LINH [1, 2].

Although MRI scan is useful for distinguishing LINH from other pituitary diseases that cause CDI, it is challenging to completely rule out the possibility of other etiologies, such as tumors because of similar clinical presentation and radiological findings. Hence, we considered performing a pituitary biopsy to make the definitive diagnosis based on the histopathological diagnosis; however, the patient declined the procedure because of its invasiveness. In this regard, the detection of anti-rabphilin-3A antibodies in the patient's serum was a valuable tool in validating the clinical diagnosis of LINH and determining a treatment and follow-up plan. Previous research by Iwama et al demonstrated that rabphilin-3A was a predominant autoantigen in LINH and that testing for anti-rabphilin-3A antibodies can differentiate LINH from other sellar/suprasellar masses with a specificity of 96.2% [4]. Anti-rabphilin-3A antibodies may prove to be a widely used diagnostic marker for LINH in future clinical settings, despite the currently unknown pathophysiologic role of these antibodies in LINH.

The benefits of GC treatment for PLH, including LINH, remain a topic of debate. Because of the rarity of this condition, there have yet to be large-scale randomized controlled trials investigating the efficacy of GC in patients with LINH. Although some cases of LINH have demonstrated radiographic regression and clinical improvement with GC treatment [7], it is generally considered a self-limiting condition that does not require such therapy [1, 2]. A recent meta-analysis on outcomes of initial strategies for PLH found that, although GC treatment was associated with a higher likelihood of anterior pituitary axis recovery and radiographic regression compared with observation, it was not significantly associated with recovery of CDI [8]. Additionally, patients who received GC treatment as an initial strategy were more likely to require additional treatment to address complications of GC treatment [8]. Given that plentiful cases of PLH, including LINH, have been reported to show spontaneous recovery with observation [2, 3, 9], the risks and benefits of GC treatment should be meticulously considered when determining an initial strategy, and observation would be appropriate, as in our case, unless the symptoms were severe or progressive [3].

Pituitary surgery is not typically indicated for patients with LINH because pituitary stalk enlargement tends to regress over time, as observed in the present case. Although patients with compromised vision or cranial nerve involvement might require surgical decompression, pituitary surgery is associated with a high risk of postoperative pituitary dysfunction [3]. Additionally, the surgery, like GC treatment, cannot preserve stalk integrity or improve CDI [1, 3, 8]. Therefore, the indications for pituitary surgery are more circumscribed compared with GC treatment and should not be performed in the absence of symptoms of mass effects, as in the current case. CDI is often permanent, resulting from pituitary stalk fibrosis, atrophy, and rarely necrosis, even with aggressive therapeutic interventions; thus, life-long desmopressin treatment is typically required in most cases of LINH [1]. In the present case, CDI persisted even after the pituitary stalk swelling regressed, with the posterior lobe bright spot remaining absent, suggesting that the transport function of the stalk was permanently impaired.

We describe the case of a patient with LINH who presented with CDI without mass effect symptoms and anterior pituitary dysfunction. As is commonly observed in cases of LINH, the autoimmune inflammatory process regressed spontaneously, whereas the stalk dysfunction persisted. In light of these observations, observation is an appropriate initial management strategy in the majority of LINH cases. Our case serves as a demonstration of the importance of using noninvasive investigations at the initial presentation to avoid unnecessary burdens in suspected cases of LINH. MRI scans of hypothalamic-pituitary regions and testing for anti-rabphilin-3A antibodies can effectively aid clinicians in diagnosing LINH without requiring invasive pituitary biopsy. However, given the inability to rule out the possibility of other diseases without biopsy [1], and that a previous report has described a case of inflammation progressing to the entire pituitary following the diagnosis of LINH [10], we should note that careful monitoring with serial hormonal assessment and neuroimaging is essential following the determination of the initial diagnosis and management plan.

## Learning Points

- Patients with CDI of unknown etiology have a good indication for the radiological evaluation of hypothalamic-pituitary regions for the diagnosis of LINH.
- The lack of a posterior pituitary bright spot presented with the stalk thickening on MRI is highly relevant to LINH.
- Because the autoimmune inflammatory process regresses spontaneously and conservative management is pertinent in most cases of LINH, appropriate clinical, hormonal, and radiological evaluation at the initial presentation is pivotal to avoid unnecessary interventions such as GC treatment or pituitary surgery.
- In combination with radiological workup, testing for anti-rabphilin-3A antibodies provides a more specific diagnosis of LINH without invasive pituitary biopsy.
- Careful follow-up with hormonal and radiological assessment after diagnosis of LINH is mandatory to affirm regression of inflammation.

## Data Availability

Data sharing is not applicable to this article as no data sets were generated or analyzed during the present study.

## Abbreviations

CDI: central diabetes insipidus
GC: glucocorticoid
LINH: lymphocytic infundibulo-neurohypophysitis
MRI: magnetic resonance imaging
PLH: primary lymphocytic hypophysitis
